# Effectiveness of a Family-based Health Promotion Intervention for Women With Prior GDM: The Face-It RCT

**DOI:** 10.1210/clinem/dgae856

**Published:** 2024-12-09

**Authors:** Karoline Kragelund Nielsen, Inger Katrine Dahl-Petersen, Dorte Møller Jensen, Peter Damm, Per Ovesen, Elisabeth R Mathiesen, Ulla Kampmann, Christina Anne Vinter, Sine Knorr, Lise Lotte Andersen, Emma Davidsen, Nanna Husted Jensen, Jori Aalders, Maja Thøgersen, Anne Timm, Henrik Støvring, Helle Terkildsen Maindal

**Affiliations:** Department of Prevention, Health Promotion & Community Care, Copenhagen University Hospital–Steno Diabetes Center Copenhagen, 2730 Herlev, Denmark; Department of Prevention, Health Promotion & Community Care, Copenhagen University Hospital–Steno Diabetes Center Copenhagen, 2730 Herlev, Denmark; Steno Diabetes Center Odense, 5000 Odense, Denmark; Department of Gynecology and Obstetrics, Odense University Hospital, 5000 Odense, Denmark; Department of Clinical Research, Faculty of Health Sciences, University of Southern Denmark, 5230 Odense, Denmark; Center for Pregnant Women with Diabetes, Department of Fertility, Gynecology and Obstetrics, Copenhagen University Hospital–Rigshospitalet, 2100 Copenhagen, Denmark; Department of Clinical Medicine, Faculty of Health and Medical Sciences, University of Copenhagen, 2200 Copenhagen, Denmark; Steno Diabetes Center Aarhus, 8200 Aarhus, Denmark; Department of Clinical Medicine, Faculty of Health, Aarhus University, 8000 Aarhus, Denmark; Department of Gynecology and Obstetrics, Aarhus University Hospital, 8200 Aarhus, Denmark; Department of Clinical Medicine, Faculty of Health and Medical Sciences, University of Copenhagen, 2200 Copenhagen, Denmark; Center for Pregnant Women with Diabetes, Department of Endocrinology and Metabolism, Copenhagen University Hospital–Rigshospitalet, 2100 Copenhagen, Denmark; Steno Diabetes Center Aarhus, 8200 Aarhus, Denmark; Department of Clinical Medicine, Faculty of Health, Aarhus University, 8000 Aarhus, Denmark; Steno Diabetes Center Odense, 5000 Odense, Denmark; Department of Gynecology and Obstetrics, Odense University Hospital, 5000 Odense, Denmark; Department of Clinical Research, Faculty of Health Sciences, University of Southern Denmark, 5230 Odense, Denmark; Steno Diabetes Center Aarhus, 8200 Aarhus, Denmark; Department of Clinical Medicine, Faculty of Health, Aarhus University, 8000 Aarhus, Denmark; Department of Gynecology and Obstetrics, Odense University Hospital, 5000 Odense, Denmark; Department of Prevention, Health Promotion & Community Care, Copenhagen University Hospital–Steno Diabetes Center Copenhagen, 2730 Herlev, Denmark; Department of Public Health, Faculty of Health, Aarhus University, 8000 Aarhus, Denmark; Department of Public Health, Faculty of Health, Aarhus University, 8000 Aarhus, Denmark; Steno Diabetes Center Odense, 5000 Odense, Denmark; Department of Clinical Research, Faculty of Health Sciences, University of Southern Denmark, 5230 Odense, Denmark; Department of Prevention, Health Promotion & Community Care, Copenhagen University Hospital–Steno Diabetes Center Copenhagen, 2730 Herlev, Denmark; Department of Public Health, Faculty of Health, Aarhus University, 8000 Aarhus, Denmark; Department of Prevention, Health Promotion & Community Care, Copenhagen University Hospital–Steno Diabetes Center Copenhagen, 2730 Herlev, Denmark; Steno Diabetes Center Aarhus, 8200 Aarhus, Denmark; Department of Biomedicine, Faculty of Health, Aarhus University, 8000 Aarhus, Denmark; Department of Prevention, Health Promotion & Community Care, Copenhagen University Hospital–Steno Diabetes Center Copenhagen, 2730 Herlev, Denmark; Department of Public Health, Faculty of Health, Aarhus University, 8000 Aarhus, Denmark

**Keywords:** gestational diabetes mellitus, diabetes prevention, family intervention, health promotion, randomized controlled trial

## Abstract

**Context:**

Gestational diabetes mellitus (GDM) increases the risk of future type 2 diabetes (T2DM), but effective and feasible interventions to reduce this risk are lacking.

**Objective:**

To evaluate the effectiveness of a family-based health promotion intervention on T2DM risk factors and quality of life among women with recent GDM.

**Design:**

Multicenter, parallel, open-label randomized controlled trial with 2:1 allocation ratio.

**Setting:**

Three sites in Denmark.

**Participants:**

Women diagnosed with GDM.

**Intervention(s):**

The intervention consisted of (1) home visits with tailored family-based counseling (2) digital health coaching, and (3) structured cross-sectoral communication.

**Main Outcome Measures:**

Primary outcomes were body mass index (BMI) and quality of life [12-Item Short-Form mental component score (SF12 MCS)] 1 year after delivery.

**Results:**

We randomized 277 women to the intervention (n = 184) or usual care group (n = 93). The intervention did not result in significantly lower BMI [−0.44 kg/m^2^; 95% confidence interval (CI) −0.98 to 0.11] or higher SF12 MCS (0.06; 95% CI −2.15 to 2.27) compared to the usual care group. A prespecified post hoc analysis demonstrated a reduced BMI in the intervention group among women with BMI ≥25 kg/m^2^ (−0.86 kg/m^2^; 95% CI −1.58 to −0.14).

Analyses of secondary and tertiary outcomes indicated significantly lower 2-hour insulin (−94.3 pmmol/L; 95% CI −167.9 to −20.6) and triglycerides (−0.18 mmol/L; 95% CI −0.30 to −0.05) levels, and odds of fasting plasma glucose ≥6·1 mmol/L (odds ratio 0.33; 95% CI 0.12 to 0.91) in the intervention group.

**Conclusion:**

The intervention did not result in lower BMI or increased quality of life but seemingly reduced other risk factors and lowered BMI in the subgroup of overweight women.

Landmark studies have documented that type 2 diabetes (T2DM) can be prevented or at least postponed in onset (1, 2). The Diabetes Prevention Program has shown that a substantial risk reduction is also obtainable among women with prior gestational diabetes mellitus (GDM)—one of the strongest predictors of T2DM (3). However, the Diabetes Prevention Program study was performed among women with impaired glucose tolerance, who on average had a 12-year interval since their first pregnancy with a GDM diagnosis (3). Since findings from a meta-analysis suggest that the relative risk of T2DM among women with prior GDM is highest within 3 to 6 years after the GDM affected pregnancy (4), there is a strong need to identify effective prevention interventions within the first years after delivery.

Furthermore, while diabetes prevention studies showed that intensive behavioral modifications focusing on weight loss, physical activity, and diet reduce the risk of T2DM, effective translations of such interventions into everyday life have proven difficult in wider populations (5). Findings from observational studies suggest that women with prior GDM often do not engage in recommended physical activity and dietary behaviors (6, 7), and numerous studies have documented a range of barriers to such behaviors among women with prior GDM (8, 9). In addition, while the transition to parenthood is sometimes highlighted as a “teachable moment” or “window of opportunity” for behavior change, there is evidence showing that becoming a parent is associated with an adverse impact on physical activity levels, energy intake, and weight gain (10, 11). Thus, the identification of effective, acceptable, and feasible interventions remains a challenge in this group of women at very high risk of T2DM.

The Face-it intervention was developed as a family-based health promotion intervention targeting women with recent GDM and their partners. Acknowledging that barriers to healthy behaviors exist at the individual, family, and health system levels (12), the intervention was multilevel and sought to reduce the risk of T2DM and increase quality of life among women with recent GDM and their families. It promotes physical activity, healthy dietary behaviors, and breastfeeding via a focus on social support, motivation, self-efficacy, risk perception, and health literacy. This paper evaluates the effectiveness of the Face-it intervention on T2DM risk factors and quality of life in women with recent GDM 1 year after delivery.

## Materials and Methods

### Design, Study Setting, and Participants

We conducted a parallel, open-label, randomized controlled trial (RCT) at 3 sites across Denmark to assess the effectiveness of the Face-it intervention in women with recent GDM. The protocol has been published (13) and the trial registered at clinicaltrials.gov (NCT03997773). Recruitment began in May 2019, and the last follow-up visit was conducted in June 2023.

The 3 study sites were in the 3 largest cities in Denmark: Copenhagen, Aarhus, and Odense. At the national level, 4% to 6% of women giving birth in the study period were diagnosed with GDM (14). Danish guidelines recommend that GDM is diagnosed based on a 75 g oral glucose tolerance test (OGTT) with a 2-hour value of ≥9.0 mmol/L. A risk factor-based screening approach is employed with an OGTT being performed at 24 to 28 gestational weeks and potentially at 10 to 20 gestational weeks depending on the presence of risk factors (15). Women diagnosed with GDM are treated in the hospital setting within the tax-funded public Danish health system. Recruitment for the trial was carried out during pregnancy by a healthcare provider at the obstetric departments at Rigshospitalet (Copenhagen), Aarhus University Hospital, and Odense University Hospital. Women were eligible for trial participation if they were attending antenatal care at 1 of the 3 recruiting hospitals, lived in the collaborating municipalities, and were able to understand and provide written informed consent in Danish. Due to the COVID-19 pandemic, the geographical eligibility criteria were extended at 2 sites (Odense and Aarhus) so that women attending the recruiting hospitals but not living in the collaborating municipalities could participate as well. If the woman had a partner, the partner was invited to participate in the trial with partner randomization following the woman's randomization allocation. The randomization was carried out 10 to 14 weeks after birth in a 2:1 ratio to the intervention or the usual care group. Results from the trial on partner outcomes will be reported in a subsequent publication. Partner participation did not affect the women's eligibility. Women with overt diabetes detected at the baseline examination, defined as fasting plasma glucose value ≥7.0 mmol/L and/or a 2-hour 75 g OGTT value of ≥11.1 mmol/L, were excluded prior to randomization.

### Ethics and Data Approvals

The study received ethical approval from the Regional Scientific Ethics Committee of the Capital Region, Danish National Committee on Health Research Ethics (H-18056033). We adhered to all EU General Data Protection Regulations and the Danish Act on supplementary provision to the regulation on the protection of natural persons regarding the processing of personal data and free movement of such data. All participants gave written informed consent.

### Randomization and Masking

Randomization was conducted in random blocks of 6/9/12/15, with separate randomization at each of the 3 trial sites. The allocation ratio was 2:1 to the intervention or the usual care group. This allocation ratio was pragmatically chosen to ensure a critical mass for running the intervention activities. The randomization procedure was generated by a statistician (H.S.) and administered via the Research Electronic Data Capture system. Allocation of participants occurred after the baseline measurements had been performed. Participants were informed about their group allocation by project staff.

### Procedures

The intervention development and content have been described in detail elsewhere (16). The intervention was coproduced using a thorough 4-stage process with representatives of the target group, healthcare providers, and other key stakeholders. In brief, it consisted of 3 major components: (1) 3 home visits with tailored family-based counseling from health visitors, (2) digital health coaching, and (3) a structured cross-sectoral communication system. Delivery of the intervention was adapted to local contexts with content tailored to the needs and resources of each individual woman and her family. The duration of the intervention was approximately 9 months, from 3 months postpartum to 1 year after delivery. Participants in the intervention group received 3 home visits by a trained health visitor during which a dialogue tool called the “The Family Wheel” with 5 themes was used: (1) GDM and risk/prevention of T2DM; (2) daily routines; (3) food and meals; (4) exercise/movement; and (5) family, friends, and network. In addition, the participants received digital health behavior coaching from a health coach via the Liva Healthcare digital platform (livahealthcare.com), which also contained features such as goal setting. The Liva platform and coaching were adapted to the Face-it study population. The health visitors and coaches received continuous education, training, and supervision (16). Finally, information about the GDM diagnosis was communicated from the obstetric departments to health visitors.

Due to the COVID-19 pandemic and a 2-month national strike among health visitors affecting 1 of the collaborating municipalities, the delivery of the intervention was periodically adapted with online visits being offered as an alternative to the home visits.

The baseline assessment was scheduled at 10 to 14 weeks after delivery and the follow-up assessment at approximately 1 year after delivery (17). Measurements were performed by trained staff at the Steno Diabetes Centers in Copenhagen and Odense and Aarhus University Hospital. Self-administered electronic questionnaires were used to obtain data on sociodemographics, use of glucose-lowering medication, family history of diabetes, tobacco use, physical activity, diet, breastfeeding, quality of life, and mental health. Information on prepregnancy body mass index (BMI), parity, and insulin use during pregnancy was gathered from medical birth records.

Participants attended the assessment fasting and underwent a standard 75 g OGTT with blood samples taken at 0 and 120 minutes. Insulin levels were analyzed using sandwich chemiluminescence immunoassay technique with automated equipment. Height was measured without shoes at baseline using a stadiometer (SECA217). Weight and body fat (percent) were measured using the InBody 270 with the participant wearing no shoes and light clothes. Waist circumference was measured halfway between the lowest point of the costal margin and the highest point of the iliac crest. Hip circumference was measured at the level of the greater femoral trochanter. Both waist and hip circumference were measured to the nearest 0.1 cm. Blood pressure was measured in a sitting position after a 5-minute rest using the Microlife BP A3L comfort. The average value of 3 measurements was used.

During the COVID-19 pandemic, a wider period for attending the baseline examination (up to 21 weeks after delivery) and no time limit for participation in the follow-up examination was implemented. This was done to allow as many participants as possible to attend the examinations during the recurrent lockdowns in Denmark in the period 2020 to 2022 and thus to secure appropriate medical and ethical treatment of participants.

### Outcomes

The trial had 2 prespecified primary outcomes: BMI, which was selected as a primary risk factor for T2DM, and 12-Item Short-Form (SF12) version 2 mental component score (MCS) (range 0-100) as an indicator of quality of life.

Secondary outcomes were fasting plasma glucose, high-density lipoprotein (HDL) cholesterol, triglycerides, diet quality measured by the Dietary Quality Scale-2017 score (1-9 scale), moderate to vigorous physical activity measured using the International Physical Activity Questionnaire—short form (IPAQ-SF), self-perceived health (SF12), perceived stress [Perceived Stress Scale (PSS)] (range 0-40), well-being measured using the World Health Organization-Five Well-Being Index (WHO-5) (range 0-100), and anxiety measured using the generalized anxiety disorder score (GAD-7) (range 0-21).

The tertiary outcomes included various clinical measures: waist and hip circumference, body fat percent, weight, and weight change percent, fasting insulin, 2-hour insulin and glucose, hemoglobin A1c (HbA1c), fasting plasma glucose ≥6.1 mmol/L (110 mg/dL), 2-hour glucose ≥7.8 mmol/L (199 mg/dL), homeostatic model assessment for insulin resistance, homeostatic model assessment for beta-cell function, systolic and diastolic blood pressure, total cholesterol, and low-density lipoprotein (LDL). In addition, tertiary outcomes also included SF12 physical component score (PCS) (range 0-100), minutes per day spent on walking (IPAQ-SF), and breastfeeding duration.

### Statistical Analysis

The sample size calculation was based on individual changes in BMI at the 1-year follow-up and comparison of the mean changes between the 2 groups. We expected a difference of −1.0 kg/m^2^ in the intervention group relative to the usual care group and a SD of individual changes of 2.5 kg/m^2^ at follow-up. With a randomization procedure of 2:1, an 80% power, and type 1 error of 5% (2-sided), the estimated sample size was 225 women at follow-up. Allowing for around 1/3 to dropout, we recruited 330 women with GDM during pregnancy. Women who were pregnant at follow-up were excluded from analyses.

Data were analyzed according to intention-to-treat and an a priori specified statistical analysis plan. We used mixed (continuous outcomes) and generalized linear mixed (binary outcomes) models to assess differences in outcomes at follow-up. The models included the study site as a random effect and were adjusted for the outcome measure at baseline. For breastfeeding duration, we used a log-Normal model with interval censoring and with robust variance estimates allowing for clustering by site. A hierarchical testing procedure with 2 separate hierarchies (1 for each primary outcome) was planned a priori to control for type 1 error rate for tests. A 2-sided *P*-value <.05 and Wald-based 95% confidence intervals (CIs) were used to assess statistical significance and support for the tested hypotheses. IPAQ scores were analyzed after a log-transformation to satisfy model requirements regarding normality of residuals.

We also conducted subgroup analyses according to baseline BMI. In addition, a per-protocol analysis was performed to assess the effect of the intervention when received as intended. In these analyses, we defined 3 subgroups based on received intervention dose: (1) 3 home visits and ≥9 contacts in the Liva app; (2) ≥2 home visits and ≥1 contact in the Liva app (but less than 3 home visits and ≥9 contacts); and (3) less than this, ie, 0 to 1 home visit and/or no contacts in the Liva app. To further assess attrition bias due to loss to follow-up, we examined the distribution of missing data between the randomization groups and carried out worst-case and best-case scenario analyses to assess the impact of missing data (data not shown). We further conducted a sensitivity analysis where we excluded participants reporting use of glucose-lowering medication at baseline (data not shown).

Data management was performed in SPSS version 29.0. Statistical analyses were performed using SAS Enterprise Guide 8.3 and Stata SE 18.

## Results

**Figure 1.**
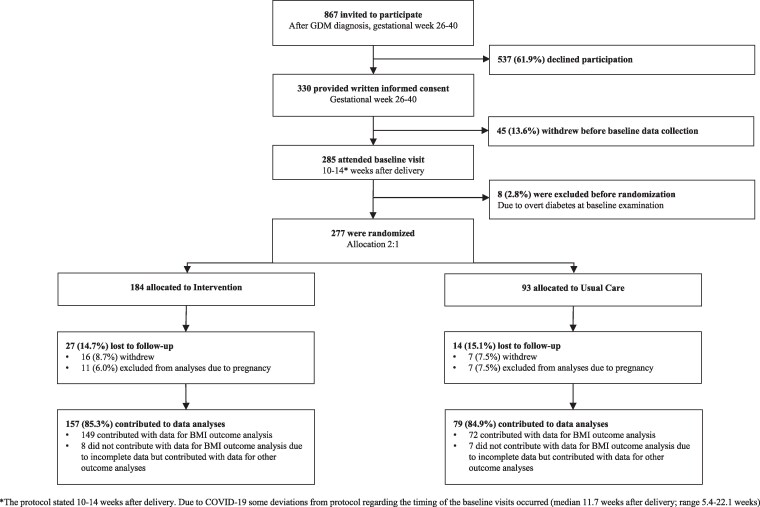
Study flowchart.

Between May 16, 2019, and June 29, 2022, 330 women were recruited during pregnancy (Fig. 1). A total of 285 women attended the baseline visit after delivery. Eight women (2.8%) were excluded at baseline due to overt diabetes, and we therefore randomized 277 women of whom 184 (66.4%) were allocated to the intervention group and 93 (33.6%) to the usual care group. The 2 groups were comparable on all measures at baseline, except women in the usual care group were a bit younger, were less likely to have been on insulin treatment during pregnancy, had slightly higher median HDL, and were more likely to breastfeed (Table 1).

**Table 1.** Baseline characteristics of the Face-it participants according to randomization group (n = 277)

Characteristics	Intervention group (n = 184)	Usual care group (n = 93)
Age at baseline (years)	33.0 (29.0-37.0)	31.0 (29.0-34.0)
Has a partner (%)	171 (92.9)	89 (95.7)
Partner participating in the Face-it trial (%)	130 (70.7)	63 (67.7)
Site (%)		
Copenhagen	32 (17.4)	18 (19.4)
Aarhus	66 (35.9)	33 (35.5)
Odense	86 (46.7)	42 (45.2)
Country/region of birth*^a^* (%)		
Denmark	147 (79.9)	70 (76.1)
Europe	14 (7.6)	6 (6.5)
Asia	14 (7.6)	10 (10.9)
Other	9 (4.9)	6 (6.5)
Low education*^a^* (%)	34 (18.5)	17 (18.5)
Employed*^a^* (%)	136 (73.9)	63 (68.5)
On maternity leave*^a^* (%)	182 (98.9)	91 (98.9)
Current tobacco use*^a^* (%)	9 (4.9)	2 (2.2)
First-degree family history of diabetes*^a^* (%)	126 (68.5)	64 (69.6)
Primiparous (%)	99 (53.8)	56 (60.2)
Prepregnancy BMI (kg/m*^2^*)*^b^*	26.7 (23.0-30.4)	27.4 (23.7-30.5)
Insulin use during pregnancy	46 (25.0%)	14 (15.1%)
Use of glucose-lowering medication at baseline*^a^*	0 (0.0%)	1 (1.1%)
Time from delivery to baseline (weeks)	11.7 (10.7-13.4)	11.3 (10.4-12.7)
BMI (kg/m*^2^*)	27.7 (23.9-31.3)	27.9 (24.8-31.1)
Weight (kg)	73.5 (64.6-85.4)	76.7 (67.0-86.5)
Waist circumference (cm)*^a^*	88.7 (81.4-98.4)	89.0 (82.1-96.0)
Hip circumference (cm)*^a^*	105.5 (99.0-113.2)	107.8 (100.8-114.5)
Body fat (%)	38.0 (±7.7)	38.8 (±7.1)
Fasting glucose (mmol/L)*^c^*	5.2 (4.8-5.4)	5.1 (4.9-5.5)
2-hour glucose (mmol/L)*^b^*	6.1 (5.2-7.2)	6.2 (5.1-7.0)
HbA1c (mmol/L)*^c^*	35.0 (32.0-37.0)	35.0 (33.0-37.0)
Fasting plasma glucose ≥6.1 mmol/L*^d^* (%)	13 (7.1)	8 (8.7)
2-hour glucose ≥7.8 mmol/L*^b^* (%)	21 (11.5)	15 (16.5)
Fasting insulin (pmol/L)*^b^*	57.5 (38.0-82.0)	55.0 (34.0-84.0)
2-hour insulin (pmol/L)*^b^*	265.0 (170.0-406.0)	281.5 (165.0-367.5)
HOMA-IR*^b^*	1.82 (1.22-2.87)	1.81 (1.11-2.95)
HOMA-Β*^b^*	103.4 (71.0-135.7)	94.3 (61.2-142.6)
Total cholesterol (mmol/L)*^c^*	4.9 (4.3-5.5)	4.8 (4.4-5.3)
HDL cholesterol (mmol/L)*^c^*	1.46 (1.30-1.71)	1.60 (1.40-1.80)
LDL cholesterol (mmol/L)*^c^*	2.90 (2.40-3.40)	2.90 (2.40-3.30)
Triglycerides (mmol/L)*^b^*	1.00 (0.70-1.30)	0.88 (0.64-1.25)
Systolic blood pressure (mmHg)	116 (108-123)	113 (108-123)
Diastolic blood pressure (mmHg)	77 (71-83)	75 (70-82)
SF12 MCS*^a^*	50.1 (44.0-54.8)	49.4 (44.2-54.4)
SF12 PCS*^a^*	54.4 (48.3-57.2)	54.7 (50.4-57.6)
Self-perceived health, excellent/very good*^a^* (%)	105 (57.1)	43 (46.7)
PSS*^a^*	13.0 (10.0-17.0)	13.0 (10.0-16.0)
WHO-5*^a^*	64.0 (52.0-72.0)	64.0 (56.0-72.0)
GAD-7*^a^*	3.0 (1.0-5.0)	3.0 (1.0-5.0)
DQS-2017*^a^*	5.0 (4.0-6.0)	5.0 (4.0-6.0)
IPAQ-SF walk (min/day)*^e^*	59.7 (28.5-120.3)	55.7 (30.0-90.0)
IPAQ-SF moderate to vigorous (min/day)*^d,e^*	57.4 (12.8-149.9)	63.4 (21.5-149.9)
Breastfeeding	144 (78.3%)	86 (92.5%)

Abbreviations: BMI, body mass index; DQS, Dietary Quality Scale; GAD-7, Generalized Anxiety Disorder 7 scale; HbA1c, hemoglobin A1c; HDL, high-density lipoprotein; HOMA-B, homeostatic model assessment for beta-cell function; HOMA-IR, homeostatic model assessment of insulin resistance; IPAQ-SF, International Physical Activity Questionnaire—short form; IQR, interquartile range; LDL, low-density lipoprotein; MCS, mental component score; PCS, physical component score; PSS, Perceived Stress Scale; SF12, 12-Item Short-Form; WHO-5, World Health Organization-Five Well-Being Index.

Data are presented as means ± SD, median (interquartile range from 25 to 75*th* percentile), or n (%).

^*a*^Data missing for 1 woman in usual care group.

^*b*^Data on prepregnancy BMI missing for 2 women in usual care group and 1 woman in intervention group. Data on 2-hour glucose and impaired glucose tolerance missing for 2 women in usual care group and 2 women in intervention group. Data on fasting insulin missing for 2 women in usual care group and 4 women in intervention group. Data on 2-hour insulin missing for 5 women in usual care group and 1 woman in intervention group. Data on HOMA-IR and HOMA- β missing for 3 women in usual care group and 4 women in intervention group. Data on triglycerides missing for 2 women in intervention group. Data on IPAQ-SF Walk missing for 1 woman in usual care group and 3 women in intervention group.

^*c*^Data missing for 1 woman in intervention group.

^*d*^Data missing for 1 woman in usual care group and 1 woman in intervention group.

^*e*^The natural log to the IPAQ variables was used to estimate medians and 95% confidence intervals, which were then back-transformed.

Twenty-three (8.3%) participants withdrew from the study [n = 16 (8.7%) in the intervention group and n = 7 (7.5%) in the usual care group], and 18 (6.5%) were excluded from analyses due to being pregnant at follow-up [n = 11 (6.0%) in the intervention group and n = 7 (7.5%) in the usual care group]. Compared to those who completed the study, those who were lost to follow-up were less likely to be employed; more likely to use tobacco; and had poorer baseline status on most anthropometric, insulin, and glucose measurements (Table 2).

**Table 2.** Baseline characteristics of the Face-it participants according to trial completion (n = 277)

Characteristics	Lost to follow-up (n = 41)	Partial completers (n = 15)	Completers (n = 221)
Randomized to intervention group (%)	27 (65.9)	8 (53.3)	149 (67.4)
Age (years)	31.0 (29.0-36.0)	32.0 (30.0-36.0)	32.0 (29.0-36.0)
Has a partner (%)	41 (100)	14 (93.3)	205 (92.8)
Partner participating in the Face-it trial (%)	29 (70.7)	10 (66.7)	154 (69.7)
Site (%)			
Copenhagen	8 (19.5)	6 (40.0)	36 (16.3)
Aarhus	17 (41.5)	4 (26.7)	78 (35.3)
Odense	16 (39.0)	5 (33.3)	107 (48.4)
Country/region of birth*^a^* (%)			
Denmark	31 (75.6)	12 (80.0)	174 (79.1)
Europe	2 (4.9)	1 (6.7)	17 (7.7)
Asia	5 (12.2)	1 (6.7)	18 (8.2)
Other	3 (7.3)	1 (6.7)	11 (5.0)
Low education*^a^* (%)	8 (19.5)	4 (26.7)	39 (17.7)
Employed*^a^* (%)	24 (58.5)	10 (66.7)	165 (75.0)
On maternity leave*^a^* (%)	40 (97.6)	15 (100)	218 (99.1)
Current tobacco use*^a^* (%)	5 (12.2)	0 (0.0)	6 (2.7)
First-degree family history of diabetes*^a^* (%)	34 (82.9)	11 (73.3)	145 (65.9)
Primiparous (%)	26 (63.4)	10 (66.7)	119 (53.9)
Prepregnancy BMI (kg/m^2^)*^b^* (%)	28.7 (25.8-32.3)	26.4 (23.5-29.7)	26.3 (22.5-30.4)
Insulin use during pregnancy (%)	8 (19.5)	5 (33.3)	47 (21.3)
Use of glucose-lowering medication at baseline*^a^* (%)	0 (0.0)	0 (0.0)	1 (0.5)
Time from delivery to baseline (weeks)	12.6 (11.0-13.7)	11.4 (10.1-12.9)	11.6 (10.6-12.9)
BMI (kg/m^2^)	30.0 (28.0-33.8)	29.1 (24.5-32.9)	26.9 (23.9-30.5)
Weight (kg)	79.8 (72.5-91.9)	72.2 (66.8-89.7)	73.3 (64.9-83.9)
Waist circumference (cm)*^a^*	95.0 (88.0-99.0)	91.4 (81.2-102.0)	87.5 (81.0-97.4)
Hip circumference (cm)*^a^*	111.0 (103.0-116.8)	108.2 (96.5-115.5)	105.6 (98.8-113.0)
Body fat (%)	41.4 (±6.0)	38.5 (±7.5)	37.7 (±7.6)
Fasting glucose (mmol/L)*^c^*	5.4 (5.0-5.6)	5.3 (4.8-5.4)	5.1 (4.8-5.4)
2-hour glucose (mmol/L)*^d^*	6.3 (5.4-7.2)	6.7 (5.3-7.3)	6.0 (5.1-7.0)
HbA1c (mmol/L)*^a^*	35.0 (33.0-36.0)	36.0 (33.0-38.0)	34.0 (32.0-37.0)
Fasting plasma glucose ≥6.1 mmol/L*^c^* (%)	2 (4.9)	3 (20.0)	16 (7.3)
2-hour glucose ≥7.8 mmol/L*^d^* (%)	4 (9.8)	1 (6.7)	31 (14.3)
Fasting insulin (pmol/L)*^e^*	70.0 (50.0-119.0)	67.0 (31.0-94.0)	50.0 (36.0-80.0)
2-hour insulin (pmol/L)*^f^*	366.0 (207.5-675.5)	308.0 (195.0-377.0)	257.5 (167.5-369.0)
HOMA-IR*^g^*	2.46 (1.66-4.12)	2.27 (0.95-3.25)	1.63 (1.13-2.75)
HOMA-Β*^g^*	127.2 (90.5-177.9)	108.8 (61.7-142.5)	95.3 (66.1-133.2)
Total cholesterol (mmol/L)*^a^*	4.9 (4.1-5.4)	4.9 (4.2-5.4)	4.9 (4.4-5.5)
HDL cholesterol (mmol/L)*^a^*	1.40 (1.21-1.50)	1.71 (1.10-1.80)	1.50 (1.30-1.80)
LDL cholesterol (mmol/L)*^a^*	3.00 (2.40-3.30)	3.10 (2.20-3.40)	2.90 (2.40-3.40)
Triglycerides (mmol/L)*^c^*	1.10 (0.80-1.50)	0.90 (0.63-1.21)	0.92 (0.70-1.28)
Systolic blood pressure (mmHg)	117 (110-126)	108 (105-130)	114 (108-122)
Diastolic blood pressure (mmHg)	77 (72-83)	76 (72-82)	77 (70-82)
SF12 MCS*^a^*	47.2 (41.0-54.7)	49.2 (42.2-55.3)	51.0 (44.9-54.7)
SF12 PCS*^a^*	53.2 (46.3-57.0)	54.3 (52.2-57.0)	54.7 (50.1-57.4)
Self-perceived health, excellent/very good*^a^* (%)	15 (36.6)	6 (40.0)	127 (57.7)
PSS*^a^*	13.0 (9.0-18.0)	14.0 (9.0-16.0)	13.0 (10.0-16.5)
WHO-5*^a^*	64.0 (44.0-72.0)	60.0 (56.0-72.0)	64.0 (52.0-72.0)
GAD-7*^a^*	4.00 (2.00-6.00)	2.00 (0.00-4.00)	3.00 (1.00-5.00)
DQS-2017*^a^*	5.00 (4.00-6.00)	5.00 (4.00-6.00)	5.00 (4.00-6.00)
IPAQ-SF walk (min/day)*^d,h^*	64.1 (34.5-90.0)	120.3 (59.7-121.5)	59.7 (25.8-90.0)
IPAQ-SF moderate to vigorous (min/day)*^c,h^*	59.7 (17.1-179.5)	73.0 (20.1-170.7)	57.4 (17.1-133.0)
Breastfeeding (%)	29 (70.7)	15 (100.0)	186 (84.2)

Abbreviations: BMI, body mass index; DQS, Dietary Quality Scale; GAD-7, Generalized Anxiety Disorder 7 scale; HbA1c, hemoglobin A1c; HDL, high-density lipoprotein; HOMA-B, homeostatic model assessment for beta-cell function; HOMA-IR, homeostatic model assessment of insulin resistance; IPAQ-SF, International Physical Activity Questionnaire—short form; IQR, interquartile range; LDL, low-density lipoprotein; MCS, mental component score; PCS, physical component score; PSS, Perceived Stress Scale; SF12, 12-Item Short-Form; WHO-5, World Health Organization-Five Well-Being Index.

Data are presented as means ± SD, median (interquartile range from 25 to 75th percentile), or n (%)

^*a*^Data missing for 1 woman in completers group.

^*b*^Data missing for 1 woman in lost to follow-up group and 2 women in completers group.

^c^Data missing for 2 women in completers group.

^*d*^Data missing for 4 women in completers group.

^*e*^Data missing for 6 women in completers group.

^*f*^Data missing for 1 woman in lost to follow-up group and 5 women in completers group.

^*g*^Data missing for 8 women in completers group.

^*h*^The natural log to the IPAQ variables was used to estimate medians and 95% confidence intervals, which were then back-transformed.

There was no difference between the intervention and usual care groups in BMI [adjusted difference (aDiff) −0.44; 95% CI −0.98 to 0.11] and SF12 MCS (aDiff 0.06; 95% CI −2.15 to 2.27) at 1-year follow-up (Table 3). However, analyses of secondary and tertiary outcomes suggest that the intervention was associated with significantly lower 2-hour insulin (aDiff −94.3; 95% CI −167.9 to −20.6) and triglycerides (aDiff −0.18; 95% CI −0.30 to −0.05) and reduced odds of fasting plasma glucose ≥6.1 mmol/L (odds ratio 0.33; 95% CI 0.12 to 0.91) in the intervention group at follow-up. In addition, women in the intervention group had nonsignificant reductions in most of the other metabolic and anthropometric outcomes. There were no differences in mental health scores, SF12 PCS, or behavioral outcomes between the intervention and usual care groups.

**Table 3.** Primary, secondary, and tertiary outcomes in intervention and usual care groups at 1-year follow-up and adjusted differences, ORs, and MRs (n = 236)

		Intervention group (n = 157)		Usual care group (n = 79)	Intervention group vs usual care group	*P*-value
	N	Value	N	Value		
Primary outcomes						
BMI (kg/m^2^)	149	27.6 (27.3 to 28.0)	72	28.1 (27.6 to 28.5)	−0.44 (−0.98 to 0.11)	.114
SF12 MCS	156	47.7 (45.9 to 49.5)	77	47.6 (45.5 to 49.8)	0.06 (−2.15 to 2.27)	.958
Secondary outcomes						
Fasting glucose (mmol/L)	148	5.40 (5.26 to 5.54)	69	5.46 (5.31 to 5.62)	−0.07 (−0.18 to 0.05)	
HDL cholesterol (mmol/L)	148	1.35 (1.31 to 1.38)	72	1.36 (1.30 to 1.41)	−0.01 (−0.08 to 0.05)	
Triglycerides (mmol/L)	147	1.02 (0.95 to 1.09)	72	1.19 (1.09 to 1.29)	−0.18 (−0.30 to −0.05)	
Self-perceived health, excellent/very good	156	70/156 (44.9%)	77	36/77 (46.8%)	OR 0.74 (0.40 to 1.37)	
PSS	150	14.3 (13.6 to 15.0)	77	14.4 (13.4 to 15.4)	−0.07 (−1.32 to 1.18)	
WHO-5	149	56.8 (54.5 to 59.2)	76	57.5 (54.2 to 60.8)	−0.69 (−4.74 to 3.36)	
GAD-7	149	4.16 (3.58 to 4.73)	76	4.45 (3.65 to 5.25)	−0.29 (−1.28 to 0.69)	
DQS-2017	155	5.20 (5.04 to 5.36)	77	5.26 (5.03 to 5.48)	−0.06 (−0.34 to 0.22)	
IPAQ-SF moderate to vigorous (min/day)*^a^*	152	33.8 (27.4 to 41.7)	77	29.4 (21.8 to 39.3)	MR 1.16 (0.80 to 1.67)	
Tertiary outcomes						
Waist circumference (cm)	149	88.3 (87.4 to 89.3)	71	88.7 (87.3 to 90.1)	−0.40 (−2.08 to 1.29)	
Hip circumference (cm)	149	105.7 (104.5 to 106.9)	71	106.7 (105.2 to 108.1)	−0.99 (−2.51 to 0.54)	
Body fat (%)	147	36.5 (35.8 to 37.1)	72	37.1 (36.2 to 38.0)	−0.66 (−1.77 to 0.45)	
Weight (kg)	149	75.5 (74.6 to 76.4)	72	76.7 (75.4 to 77.9)	−1.2 (−2.7 to 0.4)	
Weight change (%)	149	−0.54 (−1.65 to 0.57)	72	0.84 (−0.76 to 2.43)	−1.38 (−3.33 to 0.57)	
2-hour glucose (mmol/L)	142	6.13 (5.78 to 6.48)	70	6.28 (5.87 to 6.69)	−0.15 (−0.52 to 0.22)	
HbA1c (mmol/L)	146	35.9 (34.9 to 36.8)	72	36.1 (35.1 to 37.2)	−0.27 (−0.94 to 0.39)	
Fasting plasma glucose ≥6.1 mmol/L, n (%)	148	14/148 (9.5%)	69	13/69 (18.8%)	OR 0.33 (0.12 to 0.91)	
2-hour glucose ≥7.8 mmol/L, n (%)	142	17/142 (12.0%)	70	11/70 (15.7%)	OR 0.97 (0.39 to 2.43)	
Fasting insulin (pmmol/L)	144	76.8 (68.3 to 85.4)	66	84.8 (74.1 to 95.5)	−7.97 (−18.69 to 2.76)	
2-hour insulin (pmmol/L)	138	348.4 (287.9 to 408.9)	67	442.7 (368.6 to 516.7)	−94.3 (−167.9 to −20.6)	
HOMA-IR	144	2.69 (2.37 to 3.01)	65	2.99 (2.59 to 3.40)	−0.30 (−0.71 to 0.12)	
HOMA-Β	144	122.1 (109.5 to 134.6)	65	132.3 (116.1 to 148.5)	−10.18 (−26.97 to 6.58)	
Total cholesterol (mmol/L)	148	4.41 (4.32 to 4.51)	71	4.53 (4.39 to 4.67)	−0.12 (−0.29 to 0.05)	
LDL cholesterol (mmol/L)	148	2.63 (2.55 to 2.71)	71	2.62 (2.51 to 2.74)	0.01 (−0.14 to 0.15)	
Systolic blood pressure (mmHg)	149	115 (114 to 116)	72	117 (115 to 119)	−1.86 (−4.00 to 0.29)	
Diastolic blood pressure (mmHg)	149	75 (74 to 76)	72	76 (74 to 77)	−0.97 (−2.61 to 0.67)	
SF12 PCS	156	53.6 (52.3 to 54.8)	77	53.8 (52.2 to 55.3)	−0.23 (−1.75 to 1.29)	
IPAQ-SF walk (min/day)*^a^*	150	32.8 (24.3 to 44.3)	77	29.4 (20.5 to 41.7)	MR 1.13 (0.80 to 1.57)	
Breastfeeding, duration (months)*^a,b^*	128	7.86 (5.69 to 10.84)	70	8.25 (5.10 to 13.34)	MR 0.95 (0.81 to 1.12)	

Abbreviations: BMI, body mass index; DQS, Dietary Quality Scale; GAD-7, Generalized Anxiety Disorder 7 scale; HbA1c, hemoglobin A1c; HDL, high-density lipoprotein; HOMA-B, homeostatic model assessment for Beta-cell function; HOMA-IR, homeostatic model assessment of insulin resistance; IPAQ-SF, International Physical Activity Questionnaire—short form; IQR, interquartile range; LDL, low-density lipoprotein; MCS, mental component score; MR, median ratio; OR, odds ratio; PCS, physical component score; PSS, Perceived Stress Scale; SF12, 12-Item Short-Form; WHO-5, World Health Organization-Five Well-Being Index.

The table shows the estimated means at 1-year follow-up adjusted for the baseline value or the n (%) for the intervention group and usual care group, respectively, as well as the adjusted difference between the estimated means, OR, or MR.

^*a*^The natural log to the IPAQ and breastfeeding variables was used in the model. The reported estimates are backtransformed and thus estimates median ratios between groups.

^*b*^For breastfeeding duration, we used a log-Normal model with interval censoring and with robust variance estimates allowing for clustering by site. Only participants breastfeeding at baseline assessment were included in the analysis. Breastfeeding length at baseline was included as a linear covariate after log-transformation to account for breastfeeding duration prior to the baseline.

The per-protocol analyses showed no differences in BMI or SF12 MCS at 1-year follow-up between the intervention and usual care groups within any of the 3 prespecified intervention dose strata (Table 4).

**Table 4.** Per-protocol analyses of 3 groups of intervention doses vs usual care (n = 236)

		3 home visits and ≥9 contacts in the Liva app (n = 67)	3 home visits and ≥9 contacts in the Liva app Intervention group vs usual care group	*P*-value		≥2 home visits and ≥1 contact in the Liva app (n = 43)	≥2 home visits and ≥1 contact in the Liva app Intervention group vs usual care group	*P*-value		0-1 home visit and/or no contacts in the Liva app (n = 47)	0-1 home visit and/or no contacts in the Liva app Intervention group vs usual care group	*P*-value		Usual care group (n = 79)
	N	Value			N	Value			N	Value			N	Value
Primary outcomes														
BMI (kg/m^2^)	65	27.6 (27.1 to 28.1)	−0.47 (−1.12 to 0.18)	.159	41	27.9 (27.3 to 28.4)	−0.23 (−0.97 to 0.52)	.553	43	27.5 (26.9 to 28.1)	−0.60 (−1.33 to 1.13)	.108	72	28.1 (27.6 to 28.5)
SF12 MCS	66	46.7 (44.5 to 48.9)	−0.91 (−3.59 to 1.78)	.506	43	48.1 (45.5 to 50.8)	0.51 (−2.56 to 3.57)	.745	47	48.6 (46.1 to 51.1)	0.97 (−1.98 to 3.91)	.519	77	47.6 (45.6 to 49.7)
Secondary outcomes														
Fasting glucose (mmol/L)	64	5.44 (5.28 to 5.60)	−0.02 (−0.16 to 0.12)		41	5.41 (5.23 to 5.59)	−0.06 (−0.21 to 0.10)		43	5.33 (5.15 to 5.50)	−0.14 (−0.29 to 0.01)		69	5.46 (5.31 to 5.62)
HDL cholesterol (mmol/L)	64	1.38 (1.32 to 1.44)	0.02 (−0.06 to 0.10)		41	1.35 (1.28 to 1.42)	−0.01 (−0.10 to 0.08)		43	1.29 (1.22 to 1.36)	−0.07 (−0.16 to 0.02)		72	1.36 (1.30 to 1.41)
Triglycerides (mmol/L)	63	1.01 (0.90 to 1.12)	−0.18 (−0.33 to −0.03)		41	1.04 (0.91 to 1.18)	−0.15 (−0.32 to 0.02)		43	1.00 (0.87 to 1.13)	−0.19 (−0.36 to −0.03)		72	1.19 (1.09 to 1.29)
Self-perceived health, excellent/very good	66	27/66 (40.9%)	OR 0.54 (0.26 to 1.15)		43	19/43 (44.2%)	OR 0.81 (0.35 to 1.86)		47	24/47 (51.1%)	OR 1.04 (0.46 to 2.34)		77	36/77 (46.8%)
PSS	65	14.4 (13.0 to 15.9)	0.13 (−1.42 to 1.67)		40	14.7 (13.0 to 16.5)	0.42 (−1.36 to 2.20)		45	13.4 (11.8 to 15.0)	−0.89 (−2.58 to 0.81)		77	14.3 (13.0 to 15.7)
WHO-5	65	56.2 (52.4 to 60.0)	−1.29 (−6.27 to 3.69)		40	56.2 (51.4 to 61.1)	−1.22 (−6.96 to 4.52)		44	58.2 (53.8 to 62.7)	0.78 (−4.75 to 6.30)		76	57.5 (54.1 to 60.8)
GAD-7	65	4.43 (3.50 to 5.36)	−0.06 (−1.27 to 1.16)		40	3.93 (2.75 to 5.11)	−0.56 (−1.96 to 0.85)		44	4.15 (3.07 to 5.24)	−0.34 (−1.67 to 1.00)		76	4.49 (3.67 to 5.31)
DQS-2017	65	5.37 (5.13 to 5.62)	0.12 (−0.21 to 0.45)		43	5.22 (4.92 to 5.52)	−0.04 (−0.41 to 0.34)		47	4.93 (4.65 to 5.22)	−0.32 (−0.69 to 0.04)		77	5.26 (5.03 to 5.48)
IPAQ-SF moderate to vigorous (min/day)*^a^*	65	44.7 (32.5 to 61.6)	MR 1.52 (0.99 to 2.36)		42	22.6 (15.2 to 33.8)	MR 0.77 (0.47 to 1.27)		45	33.5 (22.6 to 48.9)	MR 1.14 (0.71 to 1.84)		77	29.4 (21.8 to 39.3)
Tertiary outcomes														
Waist circumference (cm)	65	87.6 (86.1 to 89.0)	−1.14 (−3.15 to 0.87)		41	89.3 (87.5 to 91.1)	0.56 (−1.73 to 2.85)		43	88.5 (86.7 to 90.3)	−0.20 (−2.46 to 2.05)		71	88.7 (87.3 to 90.1)
Hip circumference (cm)	65	105.1 (103.7 to 106.6)	−1.56 (−3.39 to 0.27)		41	105.8 (104.1 to 107.6)	−0.83 (−2.92 to 1.26)		43	106.4 (104.7 to 108.1)	−0.28 (−2.33 to 1.77)		71	106.7 (105.3-108.1)
Body fat (%)	64	36.0 (35.0 to 36.9)	−1.16 (−2.49 to 0.17)		40	37.0 (35.8 to 38.3)	−0.09 (−1.62 to 1.43)		43	36.7 (35.5 to 37.9)	−0.45 (−1.94 to 1.05)		72	37.1 (36.2 to 38.0)
Weight (kg)	65	75.6 (74.2 to 76.9)	−1.10 (−2.90 to 0.70)		41	75.9 (74.3 to 77.6)	−0.72 (−2.78 to 1.34)		43	75.0 (73.4 to 76.6)	−1.67 (−3.70 to 0.36)		72	76.7 (75.4 to 77.9)
Weight change (%)	65	−0.57 (−2.26 to 1.11)	−1.41 (−3.73 to 0.92)		41	0.24 (−1.89 to 2.36)	−0.60 (−3.26 to 2.06)		43	−1.24 (−3.31 to 0.83)	−2.08 (−4.69 to 0.54)		72	0.84 (−0.77 to 2.44)
2-hour glucose (mmol/L)	63	6.50 (6.07 to 6.93)	0.23 (−0.21 to 0.67)		40	5.85 (5.35 to 6.35)	−0.42 (−0.92 to 0.08)		39	5.82 (5.33 to 6.31)	−0.45 (−0.95 to 0.04)		70	6.27 (5.86 to 6.68)
HbA1c (mmol/L)	63	36.3 (35.2 to 37.3)	0.14 (−0.66 to 0.95)		41	36.0 (34.8 to 37.1)	−0.15 (−1.06 to 0.77)		42	35.1 (34.0 to 36.2)	−1.00 (−1.89 to −0.11)		72	36.1 (35.1 to 37.1)
Fasting plasma glucose ≥6.1 mmol/L, n (%)	64	7/64 (10.9%)	OR 0.38 (0.11 to 1.38)		41	3/41 (7.3%)	OR 0.38 (0.09 to 1.68)		43	4/43 (9.3%)	OR 0.22 (0.04 to 1.11)		69	13/69 (18.8%)
2-hour glucose ≥7.8 mmol/L, n (%)	63	10/63 (15.9%)	1.36 (0.46 to 4.00)		40	5/40 (12.5%)	OR 1.11 (0.31 to 3.91)		39	2/39 (5.1%)	OR 0.37 (0.07 to 1.92)		70	11/70 (15.7%)
Fasting insulin (pmmol/L)	64	74.1 (63.0 to 85.3)	−10.8 (−23.6 to 2.1)		39	84.7 (71.2 to 98.1)	−0.24 (−14.9 to 14.5)		41	73.8 (60.9 to 86.7)	−11.1 (−25.5 to 3.19)		66	84.9 (74.1 to 95.8)
2-hour insulin (pmmol/L)	61	361.2 (280.6 to 441.8)	−81.5 (−170.6 to 7.62)		38	340.5 (244.6 to 436.5)	−102.2 (−204.2 to −0.11)		39	337.4 (244.8 to 430.0)	−105.3 (−205.2 to −5.42)		67	442.7 (365.7 to 519.7)
HOMA-IR	64	2.58 (2.15 to 3.00)	−0.41 (−0.91 to 0.08)		39	2.98 (2.46 to 3.49)	−0.01 (−0.59 to 0.56)		41	2.60 (2.11 to 3.09)	−0.39 (−0.95 to 0.17)		65	2.99 (2.57 to 3.41)
HOMA-Β	64	119.3 (102.4 to 136.1)	−13.2 (−33.1 to 6.8)		39	135.3 (114.8 to 155.8)	2.9 (−19.9 to 25.7)		41	114.1 (94.5 to 133.7)	−18.3 (−40.6 to 4.0)		65	132.4 (115.9 to 149.0)
Total cholesterol (mmol/L)	64	4.46 (4.32 to 4.61)	−0.07 (−0.27 to 0.13)		41	4.39 (4.21 to 4.57)	−0.15 (−0.37 to 0.08)		43	4.37 (4.19 to 4.54)	−0.17 (−0.39 to 0.06)		71	4.53 (4.39 to 4.67)
LDL cholesterol (mmol/L)	64	2.64 (2.52 to 2.77)	0.02 (−0.15 to 0.19)		41	2.61 (2.46 to 2.77)	−0.01 (−0.20 to 0.19)		43	2.62 (2.47 to 2.77)	0.00 (−0.20 to 0.19)		71	2.62 (2.50 to 2.74)
Systolic blood pressure (mmHg)	65	115 (113 to 117)	−1.72 (−4.28 to 0.84)		41	116 (114 to 119)	−0.74 (−3.65 to 2.18)		43	114 (112 to 116)	−3.12 (−6.00 to −0.25)		72	117 (115 to 119)
Diastolic blood pressure (mmHg)	65	75.2 (73.7 to 76.6)	−0.91 (−2.89 to 1.06)		41	75.6 (73.8 to 77.4)	−0.50 (−2.74 to 1.74)		43	74.6 (72.8 to 76.3)	−1.49 (−3.70 to 0.72)		72	76.1 (74.7 to 77.4)
SF12 PCS	66	54.0 (52.4 to 55.6)	0.21 (−1.65 to 2.06)		43	53.5 (51.6 to 55.4)	−0.30 (−2.39 to 1.80)		47	53.0 (51.2 to 54.8)	−0.76 (−2.78 to 1.27)		77	53.8 (52.3 to 55.3)
IPAQ-SF walk (min/day)*^a^*	63	34.5 (24.1 to 49.4)	MR 1.19 (0.79 to 1.79)		42	25.0 (16.5 to 38.1)	MR 0.86 (0.54 to 1.36)		45	38.5 (25.8 to 58.0)	MR 1.32 (0.84 to 2.08)		77	29.1 (20.9 to 40.5)
Breastfeeding, duration (months)*^a,b^*	54	7.85 (6.85 to 9.00)	MR 0.67 NA		32	7.63 (5.44 to 10.69)	MR 0.98 (0.70 to 1.37)		42	7.98 (4.95 to 12.87)	MR 0.62 NA		70	8.29 (7.66 to 8.96)

Abbreviations: BMI, body mass index; DQS, Dietary Quality Scale; GAD-7, Generalized Anxiety Disorder 7 scale; HbA1c, hemoglobin A1c; HDL, high-density lipoprotein; HOMA-B, homeostatic model assessment for beta-cell function; HOMA-IR, homeostatic model assessment of insulin resistance; IPAQ-SF, International Physical Activity Questionnaire—short form; IQR, interquartile range; LDL, low-density lipoprotein; MCS, mental component score; MR, median ratio; OR, odds ratio; PCS, physical component score; PSS, Perceived Stress Scale; SF12, 12-Item Short-Form; WHO-5, World Health Organization-Five Well-Being Index.

The table shows the estimated means at 1-year follow-up adjusted for the baseline value or the n (%) for the intervention group and usual care group, respectively, as well as the adjusted difference between the estimated means, OR, or MR.

^*a*^The natural log to the IPAQ and breastfeeding variables was used in the model. The reported estimates are back-transformed and thus estimate median ratios between groups.

^*b*^For breastfeeding duration, we used a log-Normal model with interval censoring and with robust variance estimates allowing for clustering by site. Only participants breastfeeding at baseline assessment were included in the analysis. Breastfeeding length at baseline was included as a linear covariate after log-transformation to account for breastfeeding duration prior to the baseline.

However, the prespecified subgroup analyses suggest that the intervention was effective in reducing the primary outcome BMI among women with BMI ≥25 kg/m^2^ (aDiff −0.86; 95% CI −1.58 to −0.14) but not among women with BMI <25 kg/m^2^ (aDiff 0.26; 95% CI −0.49 to 1.02) (Table 5). Among women with BMI <25 kg/m^2^, the intervention, nevertheless, resulted in significant improvements in physical activity levels (median ratio 1.99; 95% CI 1.01 to 3.90).

**Table 5.** Primary, secondary, and tertiary outcomes in intervention and usual care groups at 1-year follow-up and adjusted differences, ORs, and MRs according to baseline BMI group (n = 236)

	BMI <25 kg/m^2^ n = 78						BMI ≥25 kg/m^2^ n = 158					
		Intervention group (n = 55)		Usual care group (n = 23)	Intervention group vs Usual care group	*P*-value		Intervention group (n = 102)		Usual care group (n = 56)	Intervention group vs Usual care group	*P*-value
	N	Value	N	Value			N	Value	N	Value		
Primary outcomes												
BMI (kg/m^2^)	52	22.1 (21.7 to 22.5)	22	21.9 (21.2 to 22.5)	0.26 (−0.49 to 1.02)	.489	97	30.4 (29.9 to 30.8)	50	31.2 (30.6 to 31.8)	−0.86 (−1.58 to −0.14)	.019
SF12 MCS	55	49.4 (47.0 to 51.9)	23	46.0 (42.5 to 49.4)	3.49 (−0.25 to 7.22)	.067	101	46.6 (44.9 to 48.4)	54	48.2 (45.9 to 50.4)	−1.53 (−4.25 to 1.19)	.268
Secondary outcomes												
Fasting glucose (mmol/L)	52	5.24 (5.06 to 5.43)	21	5.33 (5.11 to 5.56)	−0.09 (−0.29 to 0.11)		96	5.48 (5.32 to 5.64)	48	5.53 (5.35 to 5.71)	−0.05 (−0.20 to 0.09)	
HDL cholesterol (mmol/L)	52	1.45 (1.36 to 1.55)	22	1.44 (1.32 to 1.56)	0.01 (−0.10 to 0.12)		96	1.29 (1.24 to 1.34)	50	1.32 (1.25 to 1.39)	−0.03 (−0.12 to 0.05)	
Triglycerides (mmol/L)	52	0.85 (0.76 to 0.93)	22	0.90 (0.77 to 1.03)	−0.05 (−0.21 to 0.11)		95	1.12 (1.02 to 1.21)	50	1.31 (1.18 to 1.44)	−0.19 (−0.35 to −0.03)	
Self-perceived health, excellent/very good	55	30/55 (54.6%)	23	14/23 (60.9%)	OR 0.70 (0.22 to 2.18)		101	40/101 (39.6%)	54	22/54 (40.7%)	OR 0.76 (0.36 to 1.59)	
PSS	55	12.5 (11.4 to 13.5)	23	14.2 (12.6 to 15.9)	−1.78 (−3.75 to 0.20)		95	15.4 (14.4 to 16.3)	54	14.4 (13.2 to 15.7)	0.94 (−0.65 to 2.52)	
WHO-5	55	59.9 (51.9 to 68.0)	23	53.7 (44.5 to 62.9)	6.24 (−0.71 to 13.2)		94	54.7 (51.8 to 57.6)	53	58.8 (54.9 to 62.6)	−4.04 (−8.89 to 0.80)	
GAD-7	55	3.36 (2.46 to 4.27)	23	4.70 (3.36 to 6.05)	−1.34 (−2.89 to 0.22)		94	4.62 (3.86 to 5.38)	53	4.35 (3.34 to 5.37)	0.27 (−1.00 to 1.53)	
DQS-2017	55	5.16 (4.94 to 5.38)	23	5.36 (5.02 to 5.70)	−0.20 (−0.60 to 0.21)		100	5.22 (5.01 to 5.44)	54	5.20 (4.91 to 5.49)	0.02 (−0.34 to 0.38)	
IPAQ-SF moderate to vigorous (min/day)*^a^*	54	41.7 (29.1 to 60.3)	23	21.1 (12.1 to 37.0)	MR 1.99 (1.01 to 3.90)		98	30.3 (23.3 to 39.3)	54	33.8 (23.8 to 47.9)	MR 0.90 (0.58 to 1.38)	
Tertiary outcomes												
Waist circumference (cm)	52	76.4 (75.2 to 77.6)	22	76.0 (74.2 to 77.8)	0.44 (−1.72 to 2.59)		97	94.4 (93.1 to 95.6)	49	95.1 (93.3 to 96.9)	−0.79 (−3.00 to 1.42)	
Hip circumference (cm)	52	97.1 (95.3 to 98.9)	22	96.5 (94.1 to 98.8)	0.61 (−1.72 to 2.94)		97	109.9 (108.8 to 111.1)	49	111.8 (110.2 to 113.4)	−1.86 (−3.84 to 0.11)	
Body fat (%)	50	28.6 (27.5 to 29.6)	22	28.1 (26.6 to 29.7)	0.43 (−1.48 to 2.33)		97	40.3 (39.5 to 41.1)	50	41.5 (40.4 to 42.7)	−1.21 (−2.60 to 0.17)	
Weight (kg)	52	61.8 (60.7 to 62.9)	22	61.0 (59.3 to 62.8)	0.77 (−1.32 to 2.87)		97	82.3 (81.0 to 83.5)	50	84.6 (82.9 to 86.2)	−2.31 (−4.30 to −0.32)	
Weight change (%)	52	−1.25 (−3.01 to 0.51)	22	−2.06 (−4.79 to 0.69)	0.81 (−2.48 to 4.09)		97	−0.33 (−1.91 to 1.25)	50	2.34 (0.25 to 4.42)	−2.67 (−5.09 to −0.24)	
2-hour glucose (mmol/L)	49	5.55 (5.23 to 5.87)	22	6.00 (5.51 to 6.48)	−0.45 (−1.04 to 0.14)		93	6.43 (5.93 to 6.92)	48	6.46 (5.90 to 7.01)	−0.03 (−0.49 to 0.43)	
HbA1c (mmol/L)	52	35.1 (34.3 to 35.9)	22	35.1 (34.1 to 36.2)	−0.07 (−1.11 to 0.98)		94	36.3 (35.0 to 37.6)	50	36.6 (35.3 to 38.0)	−0.30 (−1.15 to 0.54)	
Fasting plasma glucose ≥6.1 mmol/L, n (%)	52	4/52 (7.7%)	21	3/21 (14.3%)	Too few events*^b^*		96	10/96 (10.4%)	48	10/48 (20.8%)	OR 0.28 (0.09 to 0.92)	
2-hour glucose ≥7.8 mmol/L, n (%)	49	2/49 (4.1%)	22	4/22 (18.2%)	OR 1.10 (0.09 to 13.9)		93	15/93 (16.1%)	48	7/48 (14.6%)	OR 0.99 (0.33 to 2.93)	
Fasting insulin (pmmol/L)	52	53.1 (47.5 to 58.7)	20	53.7 (44.6 to 62.8)	−0.61 (−11.30 to 10.07)		92	90.0 (77.8 to 102.1)	46	99.2 (84.4 to 113.9)	−9.22 (−24.0 to 5.61)	
2-hour insulin (pmmol/L)	49	218.8 (187.6 to 249.9)	21	307.1 (259.5 to 354.7)	−88.3 (−145.2 to −31.4)		89	415.4 (336.2 to 494.6)	46	504.4 (406.0 to 602.8)	−89.0 (−194.2 to 16.2)	
HOMA-IR	52	1.79 (1.58 to 2.00)	20	1.85 (1.51 to 2.19)	−0.06 (−0.46 to 0.34)		92	3.18 (2.70 to 3.66)	45	3.52 (2.94 to 4.10)	−0.34 (−0.92 to 0.24)	
HOMA-Β	52	89.8 (73.4 to 106.3)	20	92.6 (72.7 to 112.6)	−2.83 (−19.6 to 14.0)		92	140.9 (127.0 to154.7)	45	150.9 (131.5 to 170.3)	−10.0 (−33.2 to 13.2)	
Total cholesterol (mmol/L)	52	4.28 (4.11 to 4.45)	22	4.25 (4.00 to 4.51)	0.03 (−0.28 to 0.34)		96	4.49 (4.37 to 4.60)	49	4.66 (4.49 to 4.82)	−0.17 (−0.37 to 0.03)	
LDL cholesterol (mmol/L)	52	2.48 (2.36 to 2.60)	22	2.39 (2.21 to 2.58)	0.09 (−0.14 to 0.31)		96	2.71 (2.60 to 2.81)	49	2.73 (2.58 to 2.88)	−0.03 (−0.21 to 0.16)	
Systolic blood pressure (mmHg)	52	111 (109 to 114)	22	112 (109 to 115)	−0.49 (−4.04 to 3.06)		97	117 (116 to 119)	50	119 (117 to 121)	−2.21 (−4.82 to 0.41)	
Diastolic blood pressure (mmHg)	52	74 (73 to 76)	22	74 (72 to 76)	0.52 (−2.17 to 3.21)		97	76 (74 to 77)	50	77 (76 to 79)	−1.62 (−3.70 to 0.47)	
SF12 PCS	55	54.5 (53.2 to 55.8)	23	54.8 (52.8 to 56.9)	−0.36 (−2.81 to 2.10)		101	53.2 (51.4 to 54.9)	54	53.3 (51.3 to 55.4)	−0.18 (−2.13 to 1.77)	
IPAQ-SF walk (min/day)*^a^*	52	41.3 (30.0 to 56.3)	23	17.5 (10.8 to 27.9)	MR 2.36 (1.34 to 4.18)		98	27.9 (20.9 to 37.3)	54	34.8 (24.3 to 49.9)	MR 0.80 (0.53 to 1.22)	
Breastfeeding, duration (months)*^a,c^*	44	7.95 (3.99 to 15.87)	21	8.14 (4.24 to 15.61)	MR 0.98 (0.74 to 1.30)		84	7.81 (6.48 to 9.41)	49	8.29 (6.88 to 10.00)	MR 0.94 NA	

Abbreviations: BMI, body mass index; DQS, Dietary Quality Scale; GAD-7, Generalized Anxiety Disorder 7 scale; HbA1c, hemoglobin A1c; HDL, high-density lipoprotein; HOMA-B, homeostatic model assessment for beta-cell function; HOMA-IR, homeostatic model assessment of insulin resistance; IPAQ-SF, International Physical Activity Questionnaire—short form; IQR, interquartile range; LDL, low-density lipoprotein; MCS, mental component score; MR, median ratio; OR, odds ratio; PCS, physical component score; PSS, Perceived Stress Scale; SF12, 12-Item Short-Form; WHO-5, World Health Organization-Five Well-Being Index.

The table shows the estimated means at 1-year follow-up adjusted for the baseline value or the n (%) for the intervention group and usual care group, respectively, as well as the adjusted difference between the estimated means, ORs, or MRs.

^*a*^The natural log to the IPAQ and breastfeeding variables was used in the model. The reported estimates are back-transformed and thus estimates median ratios between groups.

^*b*^Only 7 women had fasting plasma glucose ≥6.1 mmol/L.

^*c*^For breastfeeding duration, we used a log-Normal model with interval censoring and with robust variance estimates allowing for clustering by site. Only participants breastfeeding at baseline assessment were included in the analysis. Breastfeeding length at baseline was included as a linear covariate after log-transformation to account for breastfeeding duration prior to the baseline.

## Discussion

This family-based health promotion intervention did not result in significant differences between randomization groups in either of the 2 primary outcomes (ie, BMI and SF12 MCS) among women with recent GDM. However, despite being a relatively low-intensity intervention, it did seemingly result in improvements in secondary and tertiary outcomes relevant to T2DM risk: 2-hour insulin, triglycerides, and fasting plasma glucose ≥6.1 mmol/L. Our study was powered to detect a difference in BMI of −1.0 kg/m^2^ between the 2 groups at follow-up. It is noteworthy that a third of study participants had a baseline BMI <25 kg/m^2^ and an estimated mean BMI ∼22.0 kg/m^2^ at follow-up. When evaluating the effect of the intervention stratified according to baseline BMI, the intervention group had significantly lower BMI at follow-up compared to the usual care group among women with BMI ≥25 kg/m^2^. This suggests that while the Face-it intervention was not effective at reducing BMI and improving SF12 MCS in the full trial population, it seems to have resulted in better outcomes for some T2DM risk factors and for overweight women who had lower BMI and weight 1 year after delivery compared to the usual care group.

Previous RCTs of T2DM risk reduction comparing behavioral intervention with usual care among women with prior GDM have yielded conflicting results (18, 19). Pooling data from 13 RCTs, Retnakaran et al found that lifestyle interventions can reduce the incidence of T2DM by 24% (18). Our study does not provide evidence of the effectiveness of the intervention in reducing T2DM incidence as we did not anticipate being able to see an effect on T2DM incidence 1 year after delivery (13). Instead, we used BMI as the main risk factor for T2DM. Ukke et al pooled data from 10 studies of the effect of lifestyle interventions on BMI conducted in high-income countries among women with prior GDM and found a significant mean difference of −0.60 kg/m^2^ (95% CI −1.07 to −0.13) (20), which is in the same level of difference observed in our study.

Personalized prevention of T2DM and GDM has gained prominence in recent years, with increasing recognition that the conditions and the pathways leading to these conditions are heterogeneous (21-23). The results of our subgroup analyses support the importance of disaggregating results by a priori defined characteristics. Future trials should explore the effects of different prevention modalities according to participant characteristics, including phenotypes and biomarkers as well as social, psychological, and environmental factors.

A key hypothesis of the Face-it trial was that involvement of the woman's partner would improve trial completion and outcomes. In the Canadian Families Defeating Diabetes RCT, McManus et al found that women with prior GDM were more likely to remain in the trial if their partner was a trial participant as well (24). In the Face-it trial, we did not see any association between trial completion and having a partner participating. However, while McManus et al lost almost half of their participants to follow-up, we had about 80% of participants contributing with data. In addition, we had a much higher proportion of partners participating (approximately 70% vs 37%). Whether partner participation resulted in better outcomes for the women and whether the intervention resulted in positive outcomes for the partners is unknown for now but will be investigated to provide more insights into the potential of family-based health promotion interventions to reduce T2DM risk factors.

Our study had several strengths, including its RCT design, intention-to-treat analyses, and high retention rate. Even though the trial took place in the postpartum period where women may encounter multiple barriers to healthy behaviors (25) and during a time impacted by the COVID-19 pandemic, only 8.3% of the women withdrew from the trial. This may, at least in part, be due to the coproduced, tailored, and low-intensity intervention being acceptable to both the participants and intervention deliverers (26, 27) and that couples in the intervention group found the intervention to be useful and motivating (26). Importantly, the loss to follow-up rates were similar in the 2 groups, indicating that neither the slight differences at baseline nor the open-label feature meant the usual care group was more likely to withdraw. External validity is enhanced by the multisite design and the proximity of the trial and intervention setup to routine practice. Moreover, in contrast to several other T2DM prevention intervention studies among women with prior GDM (3, 19, 24), we did not employ eligibility criteria related to BMI or glucose tolerance. With a mean age of participants in the Face-it trial around 32 years and approximately 22% born outside of Denmark, we obtained a representative demographic group compared to the general population of women diagnosed with GDM in Denmark (17). They may, however, have been slightly healthier as they were less likely to have obesity, to have delivered preterm, and to be multiparous. Moreover, due to differences in diagnostic criteria and sociodemographic characteristics, the findings may not directly apply to settings outside of Denmark and comparable countries. Finally, the relevance of outcomes was ensured via involvement of healthcare providers, women diagnosed with GDM, and researchers in the selection process (16, 28).

Limitations of the trial include the open-label feature at both participant and data analysis levels, which was inevitable due to the nature of the intervention and the 2:1 allocation ratio. The lack of blinding may have influenced expectations, perceptions, and behaviors outside the trial and thus diluted the effect of the intervention (29). Furthermore, although our randomization procedure, using blocks and separate randomization at each site, on most accounts successfully resulted in balanced baseline characteristics among the 2 groups, it did result in imbalance for some T2DM risk factors, ie, age, insulin treatment, HDL, and breastfeeding, which may have provided the usual care group with a slightly better T2DM risk profile. Importantly, to prevent selection bias, we did ensure allocation concealment with participants and data collectors remaining unaware of group assignment until the end of the baseline visit when the randomization procedure was conducted. Our analyses followed an a priori statistical analysis plan. By following this plan, we did not adjust for the imbalance in these characteristics or the significance levels for multiple comparisons. We had some missing data and loss to follow-up. Randomization allocation does not seem to have affected retention rates, and the best- and worst-case scenario analyses suggest that missing data did not considerably impact the findings. Finally, part of the study was conducted during the COVID-19 pandemic, and due to a national lockdown, the accepted period for baseline assessment was expanded to 20 weeks after delivery resulting in a shorter intervention period for some participants. Drawing clear conclusions on the impact of the pandemic is difficult. External validity may be affected by the timing and duration of the follow-up, which may be too short for T2DM and its risk factors to manifest. In line with recommendations from meta-analyses (19, 30), we initiated the intervention around 3 months after delivery. The limited effect on BMI may be impacted by our duration of follow-up, which was less than 1 year. There is some evidence indicating that the effects of lifestyle interventions on BMI reduction in women with prior GDM may be larger with longer follow-up (19). Finally, the lack of significant effects on behavioral outcomes (physical activity, diet, and breastfeeding) among women with BMI ≥25 kg/m^2^ may be due to the measurement tools displaying limited sensitivity to detect change. The hypothesized mechanisms of change in the Face-it intervention, including the role of behavioral outcomes, will be explored further in future studies.

In conclusion, although the Face-it family-based health promotion intervention, which was developed using a coproduction process and tailored to the needs and resources of the participants, did not result in lower BMI or higher quality of life at 1-year follow-up, it did show improvements in other T2DM risk factors at follow-up. It also reduced weight and BMI among overweight women. Considering the possible improvements in other T2DM risk factors and evidence showing the intervention was acceptable (26), we plan to examine if the impact of the intervention can be sustained and whether the incidence of T2DM can be reduced in the long term.

## Data Availability

The dataset analyzed during the current study is not publicly available due to General Data Protection Regulations but participant data is available from the corresponding author upon reasonable request and with required data access agreement as per Danish legislation. Additional, related documents (protocol and statistical analysis plan) are available at clinicaltrials.gov and from the corresponding author (informed consent form in Danish).

## Clinical Trial Registration

The trial is registered at clinicaltrials.gov (NCT03997773).
